# Nanocomposites SnO_2_/SiO_2_ for CO Gas Sensors: Microstructure and Reactivity in the Interaction with the Gas Phase

**DOI:** 10.3390/ma12071096

**Published:** 2019-04-02

**Authors:** Dayana Gulevich, Marina Rumyantseva, Evgeny Gerasimov, Artem Marikutsa, Valeriy Krivetskiy, Tatyana Shatalova, Nikolay Khmelevsky, Alexander Gaskov

**Affiliations:** 1 Chemistry Department, Moscow State University, 119991 Moscow, Russia; dayana-nsu@mail.ru (D.G.); artem.marikutsa@gmail.com (A.M.); vkrivetsky@gmail.com (V.K.); shatalovatb@gmail.com (T.S.); gaskov@inorg.chem.msu.ru (A.G.); 2 Boreskov Institute of Catalysis SB RAS, 630090 Novosibirsk, Russia; gerasimov@catalysis.ru; 3 LISM, Moscow State Technological University Stankin, 127055 Moscow, Russia; khmelevsky@mail.ru

**Keywords:** nanocomposites, tin dioxide, silicon dioxide, hydrothermal synthesis, gas sensor, carbon monoxide, humidity, active surface groups

## Abstract

Nanocomposites SnO_2_/SiO_2_ with a silicon content of [Si]/([Sn] + [Si]) = 3/86 mol.% were obtained by the hydrothermal method. The composition and microstructure of the samples were characterized by EDX, XRD, HRTEM and single-point Brunauer-Emmet-Teller (BET) methods. The surface sites were investigated using thermal analysis, FTIR and XPS. It is shown that the insertion of silicon dioxide up to the value of [Si]/([Sn] + [Si]) = 19 mol.% stabilizes the growth of SnO_2_ nanoparticles during high-temperature annealing, which makes it possible to obtain sensor materials operating stably at different temperature conditions. The sensor properties of SnO_2_ and SnO_2_/SiO_2_ nanocomposites were studied by in situ conductivity measurements in the presence of 10–200 ppm CO in dry and humid air in the temperature range of 150–400 °C. It was found that SnO_2_/SiO_2_ nanocomposites are more sensitive to CO in humid air as compared to pure SnO_2_, and the sample with silicon content [Si]/([Sn] + [Si]) = 13 mol.% is resistant to changes in relative air humidity (RH = 4%–65%) in the whole temperature range, which makes it a promising sensor material for detecting CO in real conditions. The results are discussed in terms of the changes in the composition of surface-active groups, which alters the reactivity of the obtained materials.

## 1. Introduction

Due to their physicochemical properties, wide-gap semiconductor metal oxides, such as SnO_2_, ZnO, WO_3_, In_2_O_3_, are widely used as materials for resistive-type gas sensors. Among them, tin dioxide has the greatest practical application. SnO_2_ is a wide-gap n-type semiconductor (*E*_g_ = 3.6 eV at 300 K [1]) which is effectively used to detect toxicity reducing gases CO, H_2_S, NH_3_, as well as volatile organic compounds (VOCs). The main requirements for the sensor material are selectivity, high sensitivity and thermal stability. The latter property is extremely important for the long-term operation of the sensor, as well as for measurements in a dynamic temperature mode (frequently used in e-nose devices), which allows an increase of the sensor signal due to more effective desorption of the products of the redox reaction from the surface of the semiconductor oxide [2,3,4,5]. In order to avoid sintering the nanoparticles of the sensor material when operating in such temperature conditions, it is necessary to carry out post-synthetic annealing at a temperature exceeding the expected maximum operating temperature during sensor functioning. However, the specific surface area of inorganic materials decreases by ~30%–40% with an increase in heat treatment temperature for every 100 °C [6,7]. It is possible to prevent aggregation and the sintering of nanoparticles by synthesizing SnO_2_ in a polymer matrix, or using surfactants [8,9], as well as with the addition of metal oxides (Mn, Mg, Co, Ni, Zn, Ca, Ba, V, Cu, etc.) and non-metals (P, B, S). A uniform distribution of such modifiers over tin dioxide surface reduces the surface energy at SnO_2_ grain boundaries and prevent the growth of particles during heat treatment [10,11,12]. However, the introduction of the second component inevitably leads to a change in the type and concentration of active centers on the surface of SnO_2_, which affects the sensor properties. To reduce the likelihood of side reactions, it is preferable to select a modifier with low own catalytic activity. This requirement is met by amorphous SiO_2_, which is also characterized by high thermal stability (Tamman temperature is 714 °C) [13]. In a number of studies on the synthesis of SnO_2_/SiO_2_ composites of various morphologies, a decrease in the growth rate and stabilization of SnO_2_ microstructure parameters, as well as an increase in the sensor signal to acetone, ethanol, and CO in dry and moist air were observed [14,15,16,17,18,19,20,21]. The decisive factor in the synthesis of such composites is the selection of the optimal Si:Sn ratio. Since silicon dioxide is a dielectric, its excessive content can lead to a disruption of the intergranular conductivity of SnO_2_.

The aims of this work were to synthesize SnO_2_/SiO_2_ nanocomposites that are resistant to sintering during long-term high-temperature annealing, to determine the Si:Sn ratio optimal for sensor measurements, to study the effect of silicon dioxide on the microstructure parameters and the active sites on the tin dioxide surface; and, finally, to reveal the influence of these parameters on the sensor properties of SnO_2_/SiO_2_ nanocomposites in CO detection in dry and humid air. The latter is of particular interest, since our previous work showed the efficiency of using SiO_2_ layer as a passive filter to reduce the negative effect of humidity on the SnO_2_ sensor properties toward CO [22].

## 2. Materials and Methods

### 2.1. Materials Synthesis

Tin dioxide was obtained as a result of thermal decomposition of α-stannic acid gel precipitated from H_2_SnCl_6_ solution (SnCl_4_·5H_2_O, 98%, Sigma-Aldrich, Saint Louis, MO, USA) using aqueous ammonia:

(1) $$H_{2}{\mathrm{SnCl}}_{6}+6{\mathrm{NH}}_{3}\cdot H_{2}O+\left(n-4\right)H_{2}O\to {\mathrm{SnO}}_{2}\cdot nH_{2}O↓+6{\mathrm{NH}}_{4}\mathrm{Cl}$$

The ammonia solution was added until reaching pH 6.5–7.0. The obtained α-stannic acid gel was separated by centrifugation and washed several times from the chloride ions with deionized water until the beginning of the peptization process, then with NH_4_NO_3_ (99%, Sigma-Aldrich) solution. After a negative reaction with AgNO_3_, the precipitate was dried at 50 °C for 24 h and the xerogel of β-stannic acid, SnO_2_ *x*H_2_O, was obtained.

Tetraethoxysilane (TEOS) (98%, Sigma-Aldrich) was used as SiO_2_ precursor. At the first stage, the reaction was carried out at 25 °C for 24 h:

(2) $${\left(C_{2}H_{5}O\right)}_{4}\mathrm{Si}+4H_{2}O\to \mathrm{Si}{\left(\mathrm{OH}\right)}_{4}+4C_{2}H_{5}\mathrm{OH}$$

The hydrolysis was carried out in a reaction medium consisting of 90% ethyl alcohol, 5% water and 5% TEOS (by volume) with acetic acid added to pH = 4, so that the hydrolysis rate exceeded the polycondensation rate.

**Figure 1:**
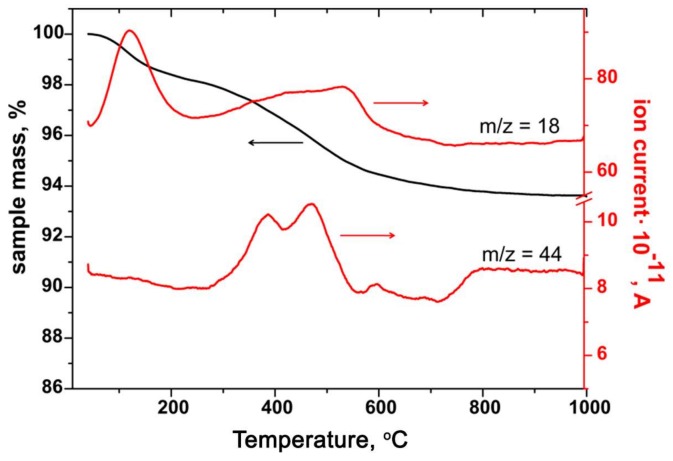
Thermogravimetric and mass spectral analysis of the SnSi49 sample before heat treatment.

To obtain the SnO_2_/SiO_2_ composites by the hydrothermal method, the SnO_2_ *x*H_2_O xerogel and an alcohol solution of Si(OH)_4_ were placed in an autoclave at a temperature of 150 °C for 24 h. The reaction was carried out with a constant stirring. The temperature was controlled using a thermocouple in contact with the lid of the autoclave. The reaction product was repeatedly washed with alcohol and water during centrifugation and dried at room temperature. According to the thermal analysis with mass spectral determination of gaseous products (CO_2_, *m*/*z* = 44; H_2_O, *m*/*z* = 18), the optimum annealing temperature of the samples was set at 600 °C, since all possible organic by-products of the TEOS hydrolysis decompose at a temperature of 500–550 °C (Figure 1). The thermal treatment was carried out in air for 24 h. The composition of the samples was pre-assigned as [Si]/([Sn] + [Si]) = 3, 13, 19, 49, and 86 mol.% (which will be referred to as SnSi3, SnSi13, SnSi19, SnSi49 and SnSi86 respectively). The designations of samples and their characteristics are given in Table 1.

### 2.2. Materials Characterization

A determination of the temperature of decomposition of the dried products of hydrothermal synthesis was made by thermal analysis with simultaneous registration of gaseous products by the mass spectral method. Thermal analysis of the samples was carried out on an STA 409 HC Luxx thermal analyzer (Netzsch-Gerätebau GmbH, Selb, Germany). Heating was performed in air flow (30 mL/min) at a rate of 10 °C/min. Mass spectral analysis of gaseous products released during the thermal decomposition of the samples was performed using a QMS 403 C Aëolos quadrupole mass spectrometer (Netzsch, Germany).

The composition of the samples was investigated by energy dispersive X-ray spectroscopy (EDX) using Zeiss NVision 40 (Carl Zeiss, Oberkochen, Germany) scanning electron microscope equipped with a X-Max detector (Oxford Instruments, Abington, UK) operated at 20 kV. The phase composition and size of the crystal grains of semiconductor materials were determined by X-ray diffraction on a DRON-4 diffractometer using monochromatic Cu Kα radiation (λ = 1.5406 Å). The survey was carried out in the range of 2θ = 10°–60° with a step of 0.1°. The crystallite size *d*_XRD_ of SnO_2_ phase was estimated from the broadening of the reflections using the Scherer formula. The phase composition was established using the STOE WinXPOW Version 1.04 program. The determination of specific surface area was performed by low-temperature nitrogen adsorption on ASAP 2020 and Chemisorb 2750 devices (Micromeritics) with subsequent analysis using the BET model.

The microstructure of nanocomposites, as well as of individual SnO_2_ and SiO_2_ oxides, was studied using high-resolution transmission electron microscopy on a JEM 2010 (JEOL, Tokyo, Japan) instrument with an accelerating voltage of 200 kV and a lattice resolution of 0.14 nm. The images were recorded using a CCD matrix of the Soft Imaging System (Mega View III, Münster, Germany). The device is equipped with an XFlash energy dispersive X-ray emission spectrometer (EDX) (Bruker, Germany) with a semiconductor Si detector with an energy resolution of 130 eV. 

The surface composition was studied using infrared spectroscopy (FTIR, Perkin Elmer Inc., Waltham, MA, USA) and X-ray photoelectron spectroscopy (XPS, Thermo Fisher Scientific, Waltham, MA, USA). The infrared (IR) spectra were recorded on a Frontier FT-IR spectrometer in the transmission mode in the wavenumbers range of 4000–400 cm^−1^ with 1 cm^−1^ step. The content of test substances in a KBr tablet (Sigma-Aldrich, “For FTIR analysis”) was 1 wt.%. The X-ray photoelectron spectra were obtained on a K-Alpha (Thermo Scientific, Waltham, MA, USA) spectrometer equipped with a monochromatic Al Kα X-ray source (*E* = 1486.7 eV). The positions of the peaks in the binding energy scale were corrected using the C1*s* peak corresponding to the carbon contamination of the surface (285.0 eV) with an accuracy of 0.1 eV. XP-spectra were fitted by Gaussian-Lorentzian convolution functions with simultaneous optimization of Shirley background parameters.

### 2.3. Study of Sensor Properties

For electrophysical and gas sensor measurements the powders of SnO_2_ and SnO_2_/SiO_2_ nanocomposites were mixed with α-terpeniol (90%, Kosher, SAFC) to form a paste and then deposited on alumina substrates with platinum contacts on the top side and platinum heater on the back side. The resulting thick films were dried at 50 °C for 24 h and annealed at 300 °C to remove residual organic binder.

The sensor properties of the materials towards CO were investigated by in situ measurements of electrical conductivity in a flow cell under controlled gas flow of 100 ± 0.1 mL/min. The measurements were carried out in the temperature interval of 400–150 °C in 50 °C steps, in the range of CO concentrations of 10–200 ppm in air. Attested gas mixture CO (0.047 ± 0.002 vol.%)/N_2_ was used as a source of carbon monoxide. Purified air was used as a background gas. The creation of gas mixtures with relative humidity (at 25 °C) RH > 1% was carried out by bubbling a part of the gas stream through a vessel with distilled water. The RH value was determined using a humidity measuring device IVTM-7 (Practic-NC, Zelenograd, Russia). The measurements were carried out under conditions of cyclic changes in the gas phase composition (three cycles for each temperature). The duration of measurements in the presence of CO and in pure air was 15 min. The magnitude of the sensor response (*S*) was calculated as *S* = *R*_air_/*R*_gas_, where *R*_air_—resistance of the sample in air, and *R*_gas_—resistance in the presence of CO.

## 3. Results and Discussion

**Figure 2:**
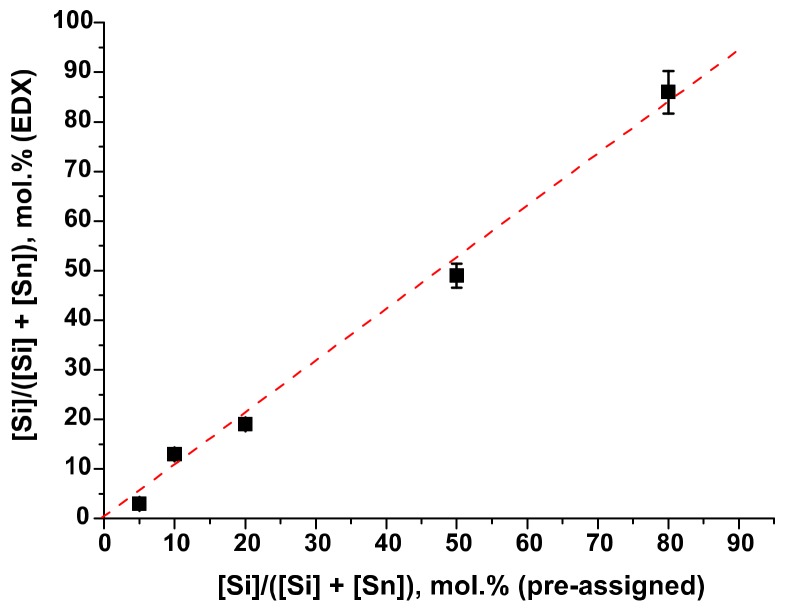
Correlation between silicon content in SnO_2_/SiO_2_ nanocomposites determined by EDX and pre-assigned during the synthesis.

**Figure 3:**
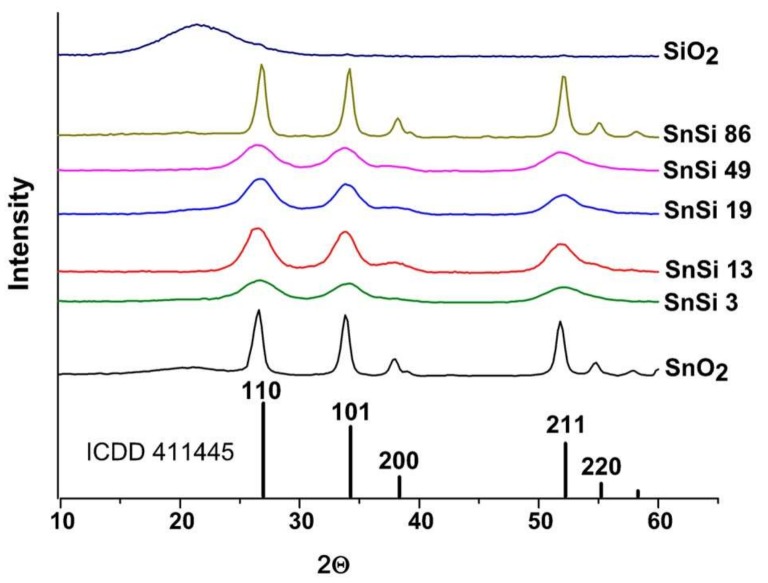
XRD patterns of nanocrystalline SnO_2_, SiO_2_ and SnO_2_/SiO_2_ nanocomposites. Vertical lines correspond to the ICDD 41-1445 reference (SnO_2_ cassiterite).

The composition of SnO_2_/SiO_2_ samples determined by the EDX method is in good agreement with the Si/Sn ratio pre-assigned during the synthesis (Figure 2, Table 1). X-ray diffraction patterns of SnO_2_/SiO_2_ nanocomposites (SnSi3–SnSi86 samples) contain only reflections corresponding to the SnO_2_ phase with a cassiterite structure (ICDD 41-1445). Silicon dioxide obtained by the hydrothermal method is X-ray amorphous (Figure 3). Crystalline phases of tin silicates are not formed under synthesis conditions. When silicon is introduced into nanocomposites, the diffraction reflections of the tin dioxide phase are broadened, which indicates a decrease in the size of the SnO_2_ crystal grains. Under isothermal annealing, the presence of impurities on the surface of growing crystallites slows down their growth rate due to the so-called Smith-Zener diffusion drag [23], according to which the maximum crystal grain size is determined by the volume fraction and particle size of another phase (including amorphous) segregated on the surface growing crystallites.

Since the key stages in the interaction of the sensor material with the gas being determined are adsorption and redox reactions on the surface of the semiconductor oxide, the high value of the specific surface area is the most important characteristic of the sample. With an increase in the annealing temperature from 300 to 600 °C, the specific surface area of SnO_2_ decreases from 90–100 to 15–20 m^2^/g, respectively [24]. At the same time a high annealing temperature allows one to obtain thermally stable sensor materials for which long-term measurements don’t lead to sintering and coarsening of nanoparticles. The tendency to a sharp decrease in the specific surface area of pure SnO_2_ is also retained in the case of hydrothermal treatment of the oxide before annealing. 

**Figure 4:**
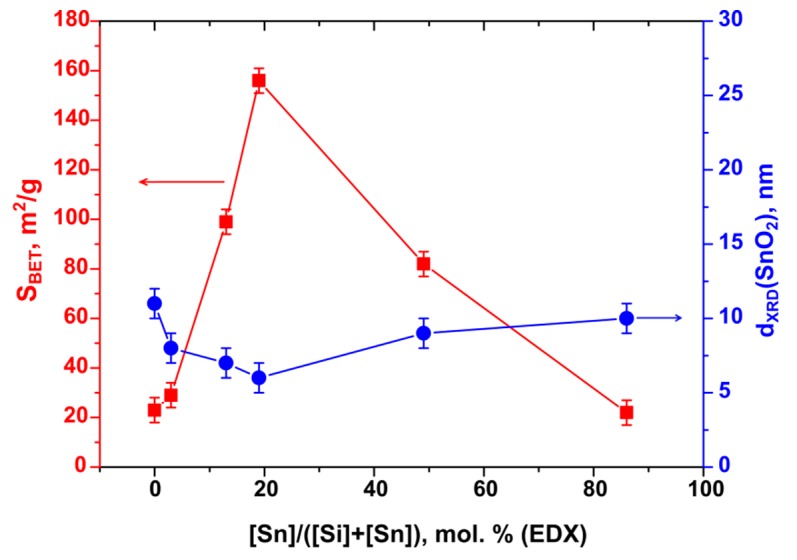
Specific surface area *S*_BET_ and SnO_2_ crystallite size *d*_XRD_ depending on silicon content in SnO_2_/SiO_2_ nanocomposites annealed at 600 °C.

**Figure 5:**
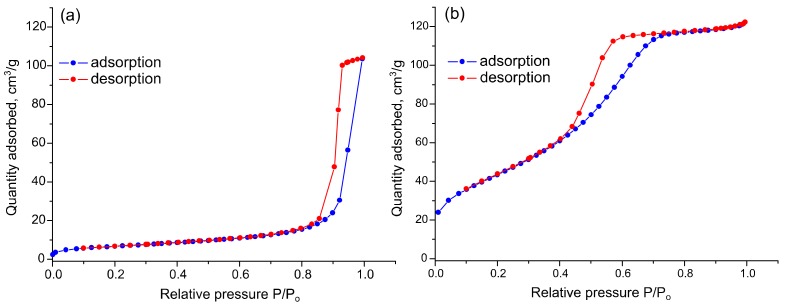
N_2_ adsorption and desorption curves of nanocrystalline SnO_2_ (**a**) and SnSi19 nanocomposite (**b**).

From the SnO_2_ crystallite size calculated by Sherer formula it can be concluded that the addition of SiO_2_ reduces the growth rate of tin dioxide nanocrystals at high annealing temperature. With the growth of silicon content in SnO_2_/SiO_2_ composites up to [Si]/([Sn] + [Si]) = 19 mol.%, an increase in the specific surface area is observed. With a further increase in [Si]/([Sn] + [Si]) ratio to 49 and 86 mol.% specific surface area of nanocomposites decreases sharply and in the latter case is almost equal to the value obtained for pure SnO_2_ (Figure 4). The N_2_ adsorption-desorption curves for pure SnO_2_ (Figure 5a) and SnSi19 nanocomposite (Figure 5b) can be attributed to type V and type IV, respectively. In both cases the hysteresis is observed, that indicates the irreversible capillary condensation. According to the IUPAC classification, in the case of SnO_2_, the hysteresis is of the H1 type, which is characteristic of a porous, spatially ordered structure that has minimal connectivity between adjacent pores. The hysteresis of the N_2_ adsorption-desorption curve of the SnSi19 nanocomposite is of the H2a type that indicates a more complex structure of mesopores characteristic for silica gels.

To explain the observed dependence of microstructure parameters on the composition of SnO_2_/SiO_2_ nanocomposites, we consider the process of nucleation of SiO_2_ on the surface of a previously formed solid phase of β-stannic acid. According to the classical nucleation theory, the work of heterogeneous nucleation is always less than the work of the formation of nuclei of a new phase through a homogeneous mechanism. Consequently, the nucleation becomes possible at low supersaturations. Thus, in the range of [Si]/([Sn] + [Si]) = 3–19 mol.%, the concentration of Si(IV) in the reaction medium is low and the supersaturation required for the formation of SiO_2_ nuclei in the volume of the reaction mixture is not reached. As a result, in the process of hydrothermal treatment SiO_2_ is formed by the mechanism of heterogeneous nucleation on the surface of SnO_2_ nanoparticles thus preventing their coarsening during subsequent heat treatment. With an increase in the concentration of silica precursor in the synthesis of SnSi49 and SnSi86 samples, the supersaturation necessary for homogeneous formation of SiO_2_ nuclei in the volume of the reaction mixture seems to be achieved. Thus, the number of SiO_2_ nuclei, covering the surface of SnO_2_, nanoparticles decreases. As a result, there is a sharp decrease in the value of the specific surface area for the nanocomposites with [Si]/([Sn] + [Si]) > 19 mol.%. The specific surface area of pure SiO_2_ obtained by the hydrothermal method from hydrolyzed tetraethoxysilan was 327 ± 5 m^2^/g. Thus, the sintering of SnO_2_ nanoparticles is responsible for reducing the specific surface area of nanocomposites. Formation of SiO_2_ fragments on the surface of tin dioxide grains by heterogeneous nucleation successfully withstands this process.

**Figure 6:**
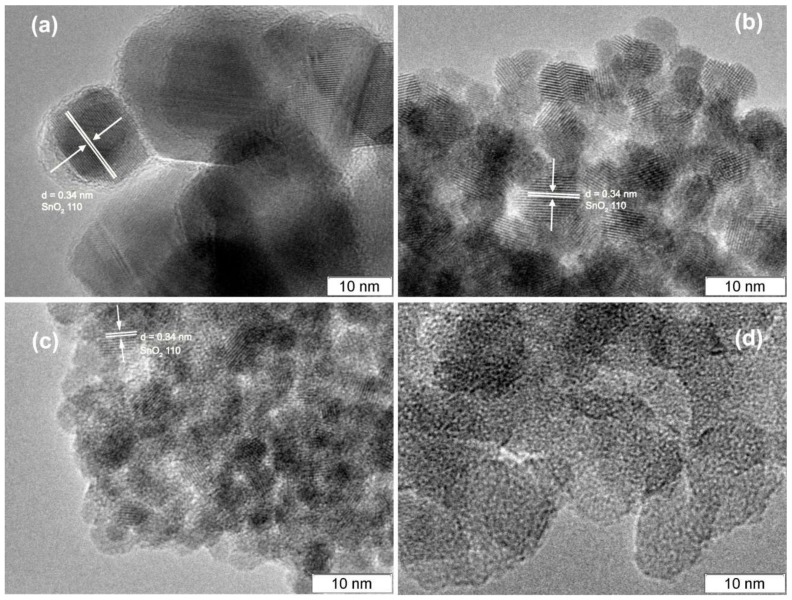
HREM images of: (**a**) SnO_2_; (**b**) SnSi13; (**c**) SnSi49; (**d**) SiO_2_.

According to the HREM image of unmodified SnO_2_ (Figure 6a), it can be concluded that tin dioxide consists mainly of large crystalline nanoparticles, while SiO_2_ is completely amorphous (Figure 6d). On the images of SnSi13 and SnSi49 samples (Figure 6b,c) one can easily highlight the crystalline phase of SnO_2_ and amorphous silica particles which are distributed fairly evenly on the surface of the semiconductor oxide. Separate large SiO_2_ aggregates are not observed even for the SnSi49 sample. The size of the crystalline particles in the composite samples decreases as compared to pure SnO_2_ and the proportion of the amorphous silica phase increases from SnSi13 to SnSi49. With an increase in the silicon concentration, the resistance of nanocomposites growths greatly due to an increase in the fraction of the dielectric SiO_2_ (Table 1). The resistance values for SnSi49 and SnSi86 samples exceed 10^11^ Ohm even at high temperature (400 °C), which makes them unsuitable for use as sensor materials.

**Figure 7:**
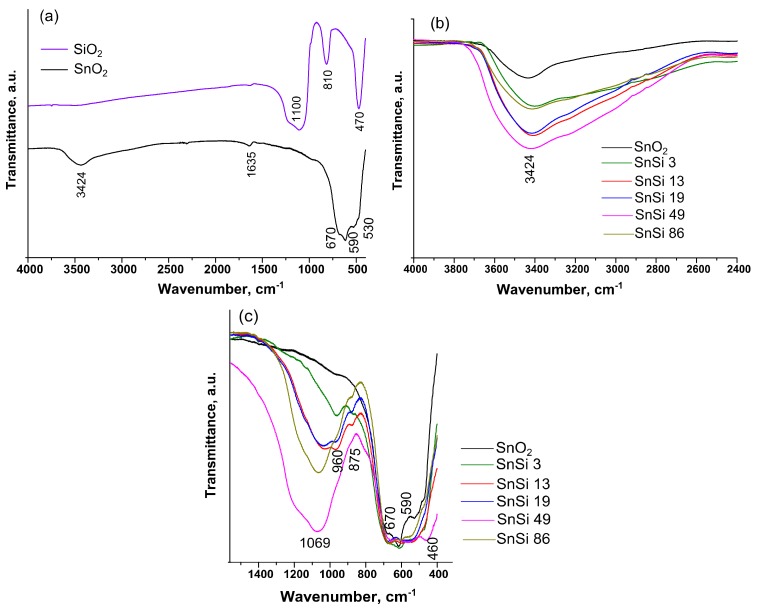
The IR spectra of the samples: (**a**) SnO_2_ and SiO_2_; (**b**) SnO_2_ and SnO_2_/SiO_2_ with the ratio [Si]/([Sn] + [Si]) = 3, 13, 19, 49, and 86 mol.% in the range of 4000–2400 cm^−1^ and (**c**) 1600–400 cm^−1^.

FTIR spectroscopy was used to investigate how the introduction of silicon dioxide affects the composition of active groups on SnO_2_ surface. The IR of unmodified tin dioxide (Figure 7a) contains stretching vibrations of O–H groups (3650–2500 cm^−1^), physically adsorbed water (1635 cm^−1^), antisymmetric vibrations of Sn–O–Sn bridge groups (670 cm^−1^), terminal Sn–OH bonds (590 cm^−1^) and symmetric vibrations of Sn–O (530 cm^−1^) [25]. On the IR spectrum of pure SiO_2_ (Figure 7a), the oscillations of hydroxyl groups and adsorbed H_2_O are practically absent. The broad absorption band with a maximum at 1100 cm^−1^ is due to the superposition of antisymmetric vibrations of Si–O–Si bridge bonds and vibrations of silanol groups (1250–870 cm^−1^). The spectrum also contains absorption bands corresponding to the symmetric vibrations of Si–O–Si (810 cm^−1^) and to the deformation vibrations of Si–O (460 cm^−1^) [26]. To estimate the change in the concentration of surface groups, the IR spectra of the composite samples were normalized to the intensity of Sn–O–Sn oscillation (670 cm^−1^) (Figure 7b,c). As the [Si]/([Sn] + [Si]) ratio increases from 3 to 49 mol.%, there is a consistent increase in the intensity of oscillations of surface hydroxyl and silanol groups. However, in the spectrum of SnSi86 sample, a noticeable decrease in the intensity of vibrations of O–H and Si–OH bonds occurs. Perhaps this is due to the condensation reaction between the silanol and hydroxyl groups on the surface of SnO_2_:

(3) $$-\mathrm{Sn}-\mathrm{OH}+\mathrm{HO}-\mathrm{Si}-\to -\mathrm{Sn}-O-\mathrm{Si}-+H_{2}O$$

This is due to the significantly larger number of formed SiO_2_ particles. The spectra of SnSi3–SnSi19 samples contain the absorbance band at 960 cm^−1^, corresponding to the antisymmetric vibrations of the [SiO_4_] group associated with Sn^4+^ (O_3_Si–Sn) [27]. The highest intensity of this absorption band is observed in the spectrum of the SnSi13 nanocomposite. This may correspond to the largest number of SiO_2_ fragments formed on SnO_2_ surface by heterogeneous nucleation. In the range of 700–400 cm^−1^, the spectra of composite samples contain all the absorption bands corresponding to the vibrations of the surface groups of individual SnO_2_ and SiO_2_.

**Figure 8:**
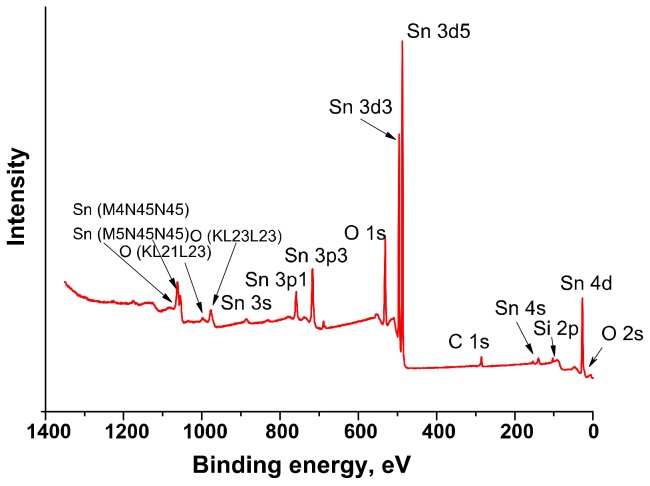
Survey X-ray photoelectron spectra of SnSi13 sample.

**Figure 9:**
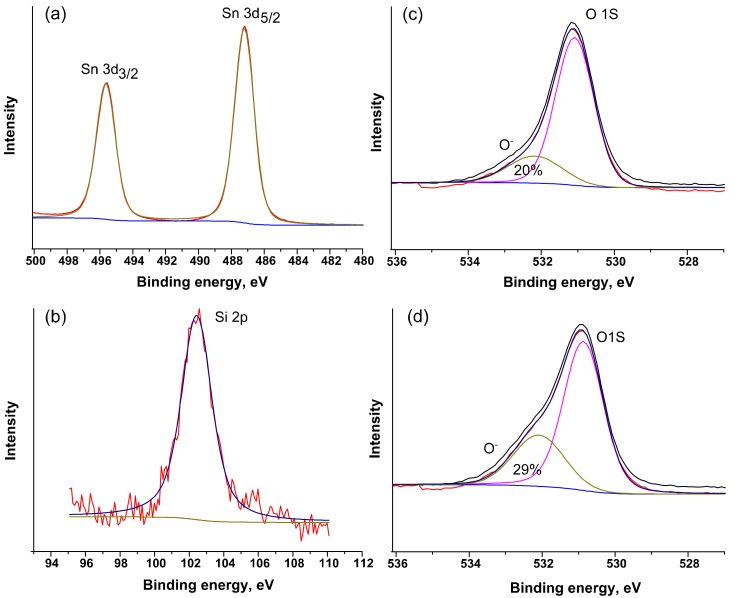
X-ray photoelectron spectra: (**a**) SnSi13 sample, Sn3*d* region; (**b**) SnSi13 sample, Si2*p* region; (**c**) SnO_2_ sample, O1*s* region; (**d**) SnSi13 sample, O1*s* region.

The main active groups on the SnO_2_ surface involved in the redox reaction in the formation of a sensor signal are chemisorbed oxygen forms:

(4) $$2{\mathrm{CO}}_{\left(\mathrm{gas}\right)}+O_{2\left(\mathrm{ads}\right)}^{-}\to 2{\mathrm{CO}}_{2\left(\mathrm{gas}\right)}+e^{-}$$

(5) $${\mathrm{CO}}_{\left(\mathrm{gas}\right)}+O_{\left(\mathrm{ads}\right)}^{-}\to {\mathrm{CO}}_{2\left(\mathrm{gas}\right)}+e^{-}$$

where CO_(gas)_ is CO molecule in the gas phase, $O_{2\left(\mathrm{ads}\right)}^{-},O_{\left(\mathrm{ads}\right)}^{-}$ are different forms of chemisorbed oxygen, CO_2(gas)_ is a product of the oxidation of CO gas desorbed into the gas phase. The change in concentration of chemisorbed oxygen in nanocomposites relative to unmodified SnO_2_ was investigated by the XPS method. Figure 8 shows the survey X-ray photoelectron spectrum of the SnSi13 nanocomposite. The spectrum contains signals of C, O, Si, Sn. The presence of peak C1*s* (285.0 eV) related with residual carbon contamination on the surface of the samples associated with analysis preparation process. The positions of Sn3*d*_5/2_ (486.6 eV) and Sn3*d*_3/2_ (497.0 eV) peaks (Figure 9a) correspond to Sn(IV) in SnO_2_ [28]. The spectrum in Si2*p* (Figure 9b) is described by a single component with an energy of 103.0 eV, corresponding to Si(IV) in SiO_2_ [28]. For nanocrystalline SnO_2_ and SnSi13 nanocomposite the XP-spectrum in O1*_S_* region has an asymmetrical shape and is described by two components (Figure 9c,d). The main peak (O1, *E* = 531.0 eV) corresponds to lattice oxygen in the structure of tin dioxide. The presence of a broad component with *E* = 532.1 eV (O2) is due to various forms of chemisorbed oxygen and hydroxyl groups. The ratio of the integral intensities of the components for pure SnO_2_ is O2/O1 = 0.25. The introduction of SiO_2_ leads to a significant increase in this value to O2/O1 = 0.36. This is consistent with the results obtained by IR spectroscopy, and may be due to an increase in the specific surface area. 

**Figure 10:**
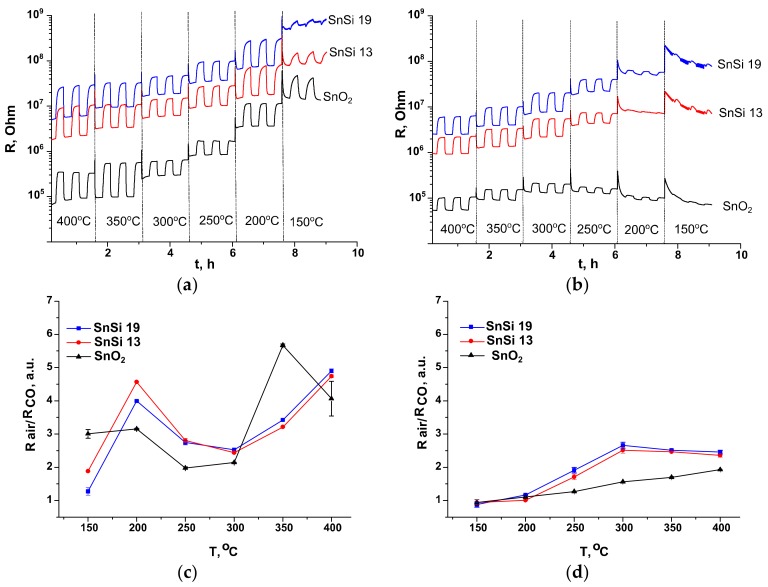
Resistance of nanocrystalline SnO_2_ and SnO_2_/SiO_2_ nanocomposites in the temperature range 400–150 °C under the periodic change of the gas phase composition: (**a**) RH = 1%; (**b**) RH = 20%. Temperature dependences of the sensor response to 100 ppm CO in air: (**c**) RH = 1%; (**d**) RH = 20%.

Sensor properties of nanocrystalline SnO_2_ and SnO_2_/SiO_2_ nanocomposites toward CO were investigated by in situ conductivity measurements. Figure 10a,b shows the change in the resistance of SnO_2_, SnSi13 and SnSi19 samples with a periodic change in the composition of the gas phase in the presence of 100 ppm CO in dry air (RH = 1%) (Figure 10a) and at relative humidity RH = 20% (at 25 °C) (Figure 10b). Tin dioxide is n-type semiconductor, therefore when interacting with a reducing gas its resistance decreases in accordance with the Equations (4) and (5).

**Figure 11:**
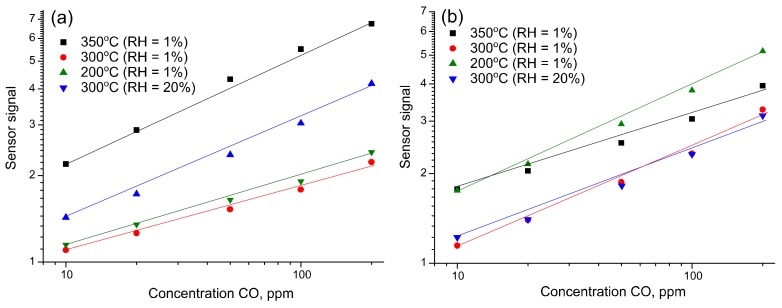

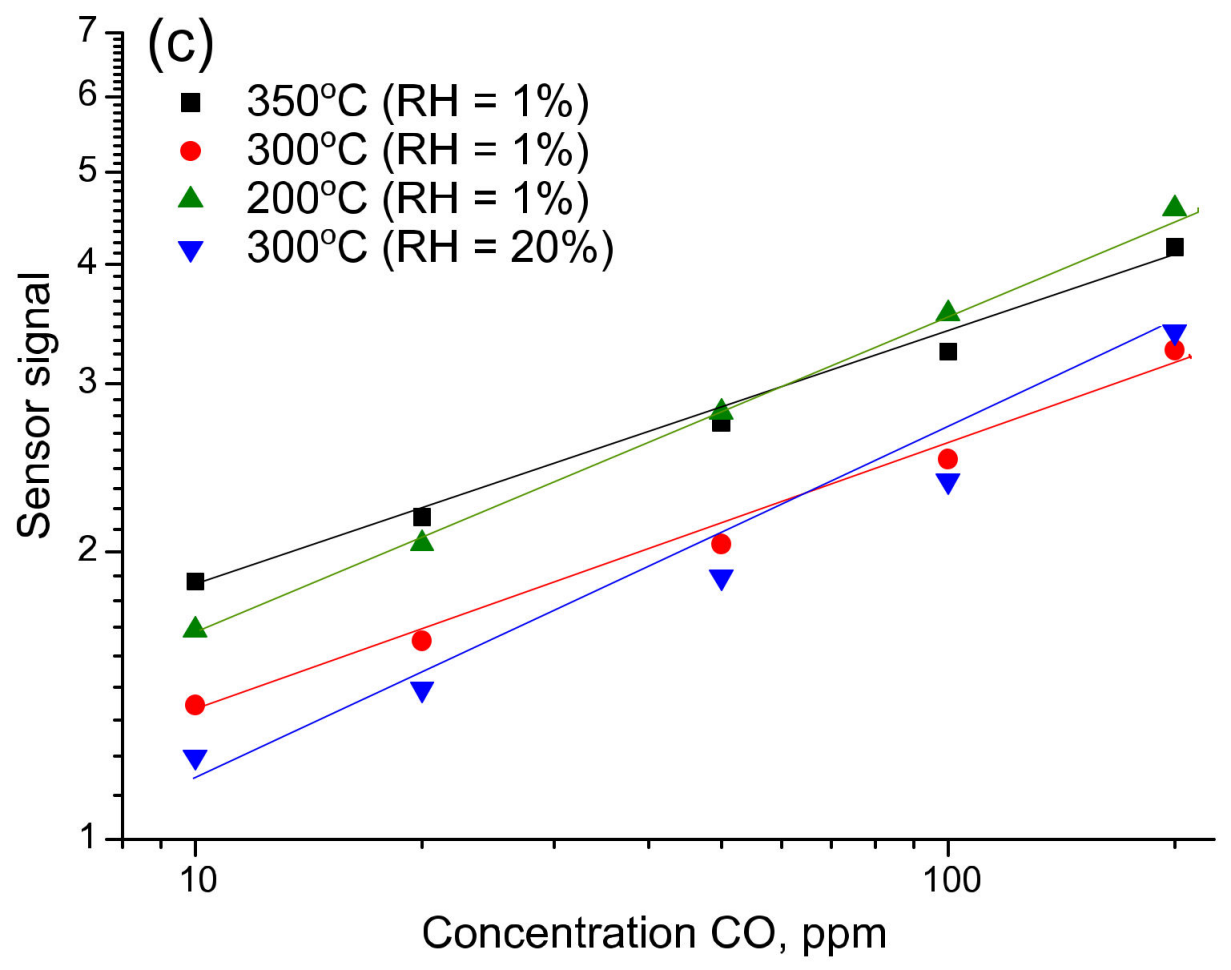
Calibration curves in double logarithmic coordinates for SnO_2_ (**a**), SnSi13 (**b**) and SnSi19(**c**).

The sensor response of all measured samples (SnO_2_, SnSi13 and SnSi19) is well reproducible. On the temperature dependence of sensor response in dry air there are two maxima: at temperatures of 350 °C for SnO_2_ and 400 °C for SnSi13, SnSi19, and also at 200 °C for all of the samples (Figure 10c). In humid air for nanocomposite samples, the maximum sensor response is observed at 300 °C, and in the case of pure tin dioxide, the magnitude of the response grows monotonically with increasing temperature up to 400 °C (Figure 10d). In general, an increase in air humidity leads to a decrease in the sensor response toward CO. However, for SnSi13 and SnSi19 nanocomposites at a measurement temperature of 300 °C, the magnitude of the signal does not depend on air humidity. Based on these results, temperatures of 350, 300, and 200 °C were selected to build the calibration curves at RH = 1% and RH = 20%. For the indicated temperatures the values of sensor response were obtained at CO concentrations of 200, 100, 50, 20, and 10 ppm CO in humid and dry air. The dependences of the sensor response *S* on the concentration of carbon monoxide *C*_CO_ correspond to a power law $S\sim C_{\mathrm{CO}}^{n}$ and are linearized in double logarithmic coordinates (Figure 11).

By calculating the average resistance value (*R*_av_) and the standard deviation (σ) of resistance in air the values of detection limits (LDL), CO for each sensor was calculated from the obtained calibration curves. The value *R*_av_/(*R*_av_–3σ) was taken as the minimum measurable sensor signal. The results are presented in Table 2.

In humid air, the sensor’s response decreases, and also the noise increases especially for the low temperature measurements (Figure 10b). However, SnO_2_/SiO_2_ samples show greater sensor response as compared to pure SnO_2_ (Figure 10d). The effect of humidity on the electrophysical properties of the samples was investigated by in situ conductivity measurements. The concentration of water vapor in the air with RH = 4%, 20%, and 65% (at 25 °C) is 0.031, 0.125, 0.625 and 2.032 vol.%, respectively. The decrease in sensor response toward CO in humid air can be explained by blocking of active centers on tin dioxide surface because of competitive adsorption of water molecules, which in turn result in the decrease in samples resistance due to the reactions:

(6) $$H_{2}O_{\left(\mathrm{gas}\right)}+2\left({\mathrm{Sn}}_{\mathrm{Sn}}+O_{O}\right)↔2\left({\mathrm{Sn}}_{\mathrm{Sn}}^{\delta +}-{\mathrm{OH}}^{\delta -}\right)+V_{O}^{2+}+2e^{-}$$

(7) $$H_{2}O_{\left(\mathrm{gas}\right)}+\left({\mathrm{Sn}}_{\mathrm{Sn}}+O_{O}\right)↔\left({\mathrm{Sn}}_{\mathrm{Sn}}^{\delta +}-{\mathrm{OH}}^{\delta -}\right)+{\mathrm{OH}}_{O}^{+}+e^{-}$$

**Figure 12:**
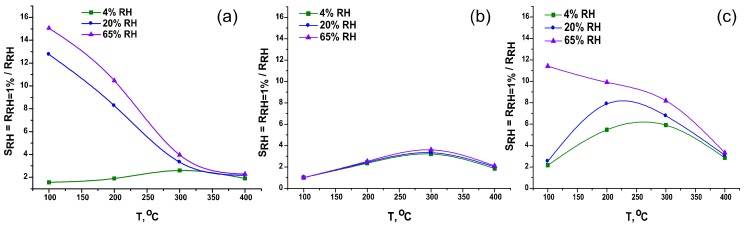
Temperature dependences of the response to water vapor of: (**a**) SnO_2_; (**b**) SnSi13; (**c**) SnSi19 at RH = 4, 20 and 65% (at 25 °C).

So, one can calculate the value of the response to water vapor as $S_{\mathrm{RH}}=\frac{R_{\mathrm{RH}=1\%}}{R_{\mathrm{RH}}}$ (Figure 12). The measurements of sensor resistances in the temperature range of 400–100 °C showed that pure SnO_2_ is the most sensitive to the content of water vapor in the air flow (Figure 12a). The response to water vapor of the SnSi13 sample (Figure 12b) over the all temperature range coincides in magnitude for all RH values, that indicates the stability of its characteristics under fluctuations in air humidity and the potential applicability of this material for CO detection in real conditions. The resistance of the SnSi19 sample varies less with increasing air humidity than pure tin dioxide (Figure 12c). 

In humid air, the greater sensor response of SnO_2_/SiO_2_ nanocomposites compared to pure SnO_2_ may be due to the tolerance (reduced sensitivity) of their electrophysical properties to hydroxyl poisoning [15]. The main cause of this effect, in our opinion, is the predominant adsorption of water molecules on the surface of SiO_2_ fragments. These fragments can act as moisture concentrators reducing hydroxyl poisoning for SnO_2_ surface, which, in its turn, is responsible for the formation of the sensor response of nanocomposites. 

**Figure 13:**
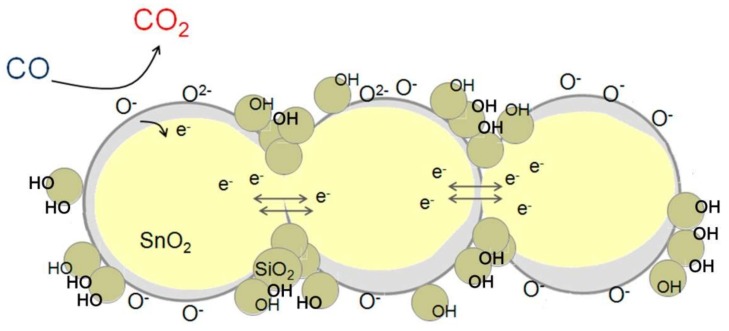
Schematic representation of the process of CO oxidationizing on the surface of SnO_2_/SiO_2_ nanocomposite under wet conditions.

The mechanism for improving the sensor characteristics of SnO_2_/SiO_2_ nanocomposites compared to pure SnO_2_ in humid air can be represented as follows (Figure 13). The SnO_2_/SiO_2_ nanocomposites contain fragments of amorphous SiO_2_, which form an interface with the SnO_2_ nanocrystals. In wet conditions, the adsorption of water vapor takes place predominantly on these SiO_2_ fragments. This reduces the number of water molecules that are adsorbed on the surface of SnO_2_. As a result, the blocking of active centers on the tin dioxide surface, which causes a decrease in the sensor signal to CO in humid air, occurs to a lesser extent. This leads to the retention of a high sensor response to CO when the sensor is operating in humid air. The sensor based on the SnSi13 nanocomposite demonstrated the greatest indifference of the sensor response to changes in air humidity.

The set of the obtained results allows us to conclude that during the synthesis of the SnSi13 nanocomposite, an optimal supersaturation of the silica precursor was created, which ensured the formation of the maximum number of SiO_2_ fragments on the SnO_2_ surface through heterogeneous nucleation. This led to the formation of such a microstructure of the nanocomposite, which allowed reducing its sensitivity to hydroxyl poisoning and increasing the sensor response to carbon monoxide in humid air.

## 4. Conclusions

SnO_2_/SiO_2_ samples were synthesized with the ratio [Si]/([Sn] + [Si]) = 0, 3, 13, 19, 49, 86, and 100 mol.%. It is shown that the addition of SiO_2_ in the range of [Si]/([Sn] + [Si]) = 3–19 mol.% allows the acquisition of materials with a high specific surface area during high-temperature annealing (600 °C) for 24h. The excess of indicated content of the dielectric in SnO_2_/SiO_2_ composition leads to an increase in the sample’s resistance above the critical value for sensor measurements. Characterization of composite materials by a complex of physicochemical methods showed that the addition of SiO_2_ at the stage of hydrothermal treatment affects not only the microstructure of the obtained samples, but also changes the composition of surface-active groups, which alters the reactivity of the obtained materials. From FTIR data it can be supposed that SnO_2_/SiO_2_ nanocomposite ([Si]/([Sn] + [Si]) = 13%) has the largest number of SiO_2_ fragments formed on SnO_2_ surface by heterogeneous nucleation. The sensor properties of SnO_2_/SiO_2_ nanocomposites toward CO were investigated in dry (RH = 1%) and humid (RH = 20%) air in the temperature range 150–400 °C. It was observed that the magnitude of the sensor response of SnO_2_/SiO_2_ nanocomposites at 300 °C does not depend on air humidity. When studying the effect of relative humidity in the range of RH = 4%–65%, it was found that the resistance of SnO_2_/SiO_2_ nanocomposite ([Si]/([Sn] + [Si]) = 13%) is the least sensitive to the RH change over the whole range of operating temperatures. This may be due to its microstructure, which reduces its sensitivity to hydroxyl poisoning. The obtained results indicate the potential applicability of this material for CO detection in real conditions.

## Figures and Tables

**Table 1:** Composition, parameters of microstructure and electrophysical properties of the obtained samples.

Sample	$\frac{\left[Si\right]}{\left[Si\right]+\left[Sn\right]}$ mol.% ^a^	*d*_XRD_ (SnO_2_), nm ^b^	*S*_BET_ ± 5 m^2^/g ^c^	*R* (100 °C), Ω ^d^
SnO_2_	0	11 ± 1	23	7.3 × 10^7^
SnSi3	3	8 ± 1	29	1.5 × 10^8^
SnSi13	13	7 ± 1	99	3.2 × 10^8^
SnSi19	19	6 ± 1	156	2.2 × 10^9^
SnSi49	49	9 ± 1	82	>10^12^
SnSi86	86	10 ± 1	22	>10^12^
SiO_2_	100	–	327	insulator

^a^: determined by EDX; ^b^: SnO_2_ crystallite size; ^c^: specific surface area; ^d^: resistance in air at 100 °C.

**Table 2:** CO detection limit (LDL) for SnO_2_, SnSi13 and SnSi19 samples, as well as the power coefficients (*n*) calculated from obtained calibration curves.

Sample	RH = 1%						RH = 20%	
	350 °C		300 °C		200 °C		300 °C	
	*n*	LDL, ppm	*n*	LDL, ppm	*n*	LDL, ppm	*n*	LDL, ppm
SnO_2_	0.457 ± 0.007	1.5	0.20 ± 0.03	8.2	0.29 ± 0.02	6.9	0.21 ± 0.04	9.0
SnSi13	0.35 ± 0.02	4.3	0.31 ± 0.04	7.2	0.58 ± 0.05	3.0	0.34 ± 0.05	7.0
SnSi19	0.34 ± 0.02	3.3	0.30 ± 0.08	5.6	0.51 ± 0.04	2.4	0.29 ± 0.03	7.8
